# Reproductive Performance of a Declining Forest Passerine in Relation to Environmental and Social Factors: Implications for Species Conservation

**DOI:** 10.1371/journal.pone.0130954

**Published:** 2015-07-14

**Authors:** Alex Grendelmeier, Raphaël Arlettaz, Michael Gerber, Gilberto Pasinelli

**Affiliations:** 1 Swiss Ornithological Institute, Sempach, Switzerland; 2 Division of Conservation Biology, Institute of Ecology and Evolution, University of Bern, Bern, Switzerland; 3 Schweizer Vogelschutz SVS/BirdLife Schweiz, Zürich, Switzerland; Institute of Ecology, GERMANY

## Abstract

Identifying factors influencing a species' ecological niche and demography is a prerequisite for species conservation. However, our understanding of the interplay between demographic rates and biotic/abiotic factors is still poor for most species of conservation concern. We evaluated relevance of eight hypotheses relating to timing of breeding, temporal nest exposure, nest concealment, topography, tree structure, predation risk and disturbance, density dependence and weather for explaining variation in reproductive performance of the declining wood warbler *Phylloscopus sibilatrix* in northern Switzerland. Reproductive performance was monitored with cameras at 136 nests from 2010 to 2012 and was associated to temporal exposure, timing of breeding and concealment of nests. Daily nest survival was positively related to the number of grass and sedge tussocks, nest concealment and nest age. Clutch size and number of fledglings decreased, the later in the season a nest was initiated. Nest survival over an average nesting period of 31 days was 46.9 ± 0.07% (mean ± SE), daily nest survival rate was 0.976 ± 0.002. As for many ground-breeding birds, nest predation was the principal cause of nest failure, accounting for 79% of all nest losses. Conservation measures should aim at increasing the area of relatively homogenous forest stands featuring suitable habitats characterized by abundant and accessible grass and sedge tussocks. In managed forests, such conditions can be found in stands of middle age (i.e. pole wood) with little to no shrub layer.

## Introduction

Population dynamics is driven by the four vital rates fertility, survival, immigration and emigration [1]. Which vital rate is most important for population dynamics remains largely species-specific. Population growth rate in many large, long-lived animal species is mostly affected by (adult) survival, whereas population growth rate in small, short-lived species is usually most strongly influenced by fertility, reproductive performance and recruitment [2]. In the case of small passerines, reproductive performance can itself be sub-divided into different components, such as clutch size, number of hatchlings, number of fledglings or nest survival. Understanding the relative contribution of each component to reproductive success and population growth is crucial, but remains poorly understood in most species [3]. Clutch size gives information about how much a female can allocate to reproduction, which may be influenced by her body condition, perceived predation risk, inter- and intraspecific competition, natural and anthropogenic disturbance, food availability, habitat suitability and interactions among these aspects [4,5,6,7]. Number of fledglings provides a reproductive performance value per individual, breeding pair or population. It further results from nestling survival, which itself depends upon weather conditions, food availability, parents’ quality, perceived predation risk and actual predation [7,8,9,10,11,12]. Nest survival thus represents an overall estimate of breeding success within a population. Examining these three components of reproductive performance in relation to potentially influential environmental factors may provide important information on an animal’s life-history [13,14,15] and should help in creating management plans for species of conservation concern. Using data from 3 years and 136 nests, this study evaluates thematically grouped hypotheses, introduced separately below, regarding the relationships between three components of wood warbler (*Phylloscopus sibilatrix*) reproductive performance–clutch size, number of fledgling and nest survival–and various environmental and social factors.

### Timing of breeding

Reproductive performance has been shown to depend on when in the season breeding is initiated [16,17,18,19,20,21,22]. Males can increase their fitness by securing high quality territories, and high-quality territories are typically among the earliest occupied in the season [23]. In many animal species, females select mates based on both specific male traits and habitat cues [24,25,26]. Females then choose the time of breeding based on environmental cues. Females should time their broods so that the peak food availability matches the highest demands of nestlings [27] and periods of increased predator abundance late in the breeding season are avoided [18]. Thus, we predicted decreased clutch sizes, number of fledglings and nest survival as the breeding season progresses.

### Temporal exposure

The relationship of nest survival with nest age appears to differ among species. While several studies have reported no difference in nest survival between incubation and nestling stage [28], others have reported either higher nest survival during incubation [29,30], higher nest survival during nestling stage [31,32] or even multimodal distributions of nest survival throughout the nesting phase [17]. Under the assumption that parental food provisioning activity increases with nest age due to increased food requirements of maturing nestlings [33], which in turn increases predation risk [29], we predicted a negative relationship between nest survival and nest age.

### Nest concealment

As nest predation is the principal cause of nest failure in many bird species, nest concealment and nest substrate choice are very important, especially for ground nesting species [34]. Some studies have found positive effects of nest concealment on reproductive performance [21,29,30,35], whereas others have not [36,37]. We predicted a positive relationship between nest survival and nest concealment.

### Topography

Exposition, elevation and inclination can be important topographical factors affecting reproductive performance via weather conditions and/or solar radiation. In relation to reproductive performance, these factors may especially influence temperature stress of incubating females and their nestlings at both low and high ambient temperatures. In temperate forest birds, reproductive performance is often increased at lower elevations due to more favorable temperatures, with a lower likelihood of freezing to death [38,39,40]. Inclined slopes exposed to the south generally receive more solar radiation than flat terrain or slopes exposed to the north. By building nests on south-east exposed slopes, high midday temperatures and/or prevailing west winds can be avoided, which may increase nestling survival [41,42]. We predicted a negative relationship of nest survival, clutch size and/or number of fledglings with elevation. Moreover, nests on slopes exposed to south-east were expected to have increased survival, larger clutches and more fledglings than nests with other expositions.

### Tree structure

Forest and tree structure can play a role in relation to daily nest survival rate, correlating with canopy cover [43] or basal area of trees [44]. As discussed in the previous paragraph, incubating females and their nests can be sensitive to temperature stress. Assuming microclimatic weather conditions are not only influenced by topographical factors but also by tree structure, we predicted a quadratic relationship between nest survival and canopy cover. Territories in open stands could be subject to solar radiation that is too high, while completely closed stands would allow almost no solar radiation and exhibit microclimatic conditions that are too humid and cool.

### Predation risk and disturbance

Nest predation is the most important cause of nest failure in most bird species worldwide, including ground nesting forest birds [45,46,47,48], and is therefore a major force driving avian demography and evolution. We investigated the relationship between reproductive performance and rodent abundance, as negative relationships between rodent and wood warbler abundances [49], as well as between settlement probability of wood warblers and rodent abundance have been found [50]. It is also known that varying rodent numbers trigger numerical and/or behavioral responses of rodent hunting predators [51]. It may be possible that birds use rodent abundance as a proxy for general predation risk. Whether through direct rodent predation or predation through rodent hunting predators, we predicted a negative relationship between nest survival and rodent abundance. Zanette et al. [7] have shown that females exposed to experimentally simulated predation risk produced smaller clutches and fewer fledglings than females not exposed to simulated predation risk. Accordingly, we predicted smaller clutches and fewer fledglings with increasing rodent abundance.

Nest predation may also increase due to edge effects connected to outer (i.e. forest edge) or inner (i.e. forestry trails) habitat edges [34,52]. Certain predators are known to use habitat edges to travel and forage [53,54]. Habitat edges can also be connected to spillover predation, a process where nest predators cross over from adjacent habitats [55]. We assessed increased predation risk connected to habitat edges with the variables “distance to closest forest edge” and “distance to closest human used trail”. However, human used trails may not only be linked to predator abundance but also to disturbance via recreational activity, which is known to have mostly negative effects on birds, especially on ground nesting species [56,57,58]. According to a review by Steven et al. [58] dealing with non-motorized disturbance on birds, 61 out of 69 studies showed negative impacts. Hiking/touring had the highest negative impact, reported in 45 out of 51 studies. Accordingly, we predicted increased nest survival, clutch sizes and number of fledglings with increased distances from forest edges and from human used trails, respectively.

### Density dependence

Previous studies have suggested that cues received from conspecifics to be just as important as or sometimes even more important than habitat quality in terms of settlement [59,60,61]. Conspecific cues may provide readily available information about a specific habitat or conspecifics themselves and if individuals subsequently decide to settle close to conspecifics, a clustering of territories and nests may result. Clustered conspecifics may also have effects on each other post settlement by influencing reproductive performance [62,63]. Territory clustering can be looked at in the context of positive density dependence or the Allee effect, a positive correlation between population density and fitness [2]. Clustering could arise due to sexual selection mechanisms that increase an individual’s fitness such as chances for extra pair parentage or due to natural selection mechanisms such as anti-predator strategies [62,63]. As territory clustering has been observed in wood warblers [64], we investigated whether reproductive performance was positively related to population density. We evaluated models including two variables related to population density, namely the distance to the closest nest and the number of nests within 300 m from a focal nest in relation to our three components of reproductive output. We predicted positive non-linear relationships [65] between population density and clutch size, number of fledglings and/or nest survival, respectively. However, negative density dependence has been shown to occur in a variety of taxa [2], and we therefore also tested for a negative relationship between population density and our three measures of reproductive performance.

### Weather

Variation in reproductive performance could also be explained by weather factors, such as ambient temperature, rainfall or solar radiation. High amounts of rainfall and low temperatures can affect food resources [22,66] and/or chick provisioning performance by parents, predator activity [67] and even parental fitness, including life span und lifetime reproductive success [68]. Rodriguez and Bustamante [69] found that nest success of the lesser kestrel (*Falco naumanni*) was positively influenced by winter rainfall but negatively by rainfall during the nestling period. Arlettaz et al. (2010b) demonstrated that, in the hoopoe (*Upupa epops*), food provisioning activity of chicks by parents was dramatically affected by adverse weather (heavy rainfall and low ambient temperature) conditions, resulting in decreased number of fledglings per brood. Thus, we predicted decreased clutch sizes, number of nestlings and nest success with increased rainfall and decreasing ambient temperature just before and/or during nesting.

## Material and Methods

### Study species and area

The wood warbler is a long-distance migratory, insectivorous forest passerine, exhibiting very little site fidelity (ring return percentages reviewed in [49]). Exhibiting great annual local population fluctuations, the species’ nomadic behavior is hypothesized to be a response to annually varying local rodent densities [49]. This species has wintering grounds in tropical Africa [70] and breeding grounds spanning northern and temperate Europe as far east as the Ural Mountains [33,71]. Breeding sites are typically occupied between April and July. Across Europe, the population trend of this species is moderately negative. Regionally, however, the species has dramatically declined, as for example in the UK, Germany or the Netherlands [72,73,74]. It is classified as “Least concern” on the current IUCN red list due to a large breeding range and high number of breeding pairs [75]. However, similar as in the aforementioned countries, the decline of this species in Switzerland is strong and the species is classified as vulnerable (VU) on the current red list of breeding birds of Switzerland. In addition, the wood warbler is considered a priority species for the Swiss species recovery program [76,77].

The study took place along northern Switzerland’s Jura mountain chain, as well as at one sitenear Lake Constance and one site in the pre-alpine valley of Glarus: 1. Bänkerjoch (Canton Aargau (AG); N 47° 26.2’ E 8° 2.1’), 2. Staffelegg (AG; N 47° 25.4’ E 8° 4.1’), 3. Blauen (Basel Landschaft (BL); N 47° 27.6’ E 7° 31.7’), 4. Dittingen (BL; N 47° 26.9’ E7° 28.8’), 5. Langenbruck (BL; N 47° 21.3’ E 7° 47.0’), 6. Lauwil (BL; N 47° 22.5 E 7° 39.7), 7 Oltingen (BL; N 47° 25.8’ E 7° 56.5’), 8. Ennenda (Glarus (GL); N 47° 2.3’ E 9° 5.0’), 9. Montsevelier (Jura (JU); N 47° 22.1’ E 7° 29.5’), 10. Belchen (Solothurn (SO); N 47° 21.7' E 7° 48.6'), 11. Erschwil (SO; N 47° 22.7 E 7° 33.3'), 12. Hochwald (SO; N 47° 27.5' E 7° 39.7'), 13. Homberg (SO; N 47° 21.5', E 7° 50.9'), 14. Kleinlützel (SO; N 47° 26.3’ E 7° 25.9’), 15. Scheltenpass (SO; N 47° 20.8’ E 7° 37.1’), 16. Gündelhart (Thurgau (TG); N 47° 38.7’ E 8° 56.4’). Using data from the common breeding bird survey (the standardized Swiss national bird monitoring program, http://www.vogelwarte.ch/en/projects/monitoring/monitoring-common-breeding-birds.html) and from the Swiss ornithological information service (casual observations of rare breeding and visiting birds, http://www.vogelwarte.ch/en/projects/monitoring/information-service-monitoring-rare-breeding-and-visiting-birds.html), regions with wood warbler occurrence in the past decade were localized and used as a rough starting point to map wood warbler territories. This approach yielded a total of 16 study areas, which range in size from 1.4 to 21 ha, in elevation from 430 to 1132 m a.s.l., and in inclination from 2 to 50 degrees. Most wood warbler territories, and hence the study areas, were located on slopes exposed to the south and consisted of deciduous and mixed-forest stands dominated by European beech (*Fagus sylvatica*), with coniferous tree species interspersed (*Picea abies*, *Abies alba*, *Pinus sylvestris*). Stands predominantly consisted of old pole wood and young timber with a relatively closed canopy and a sparse shrub layer. Generally, the field layer consisted of grass and sedges and/or herbaceous species. Several study areas had been declared as forest reserves, or were privately owned, meaning they were subjected to no or very limited forestry interventions in the past decades (personal communication of landowners and foresters).

### Field methods

Data was collected between April and September from 2010 to 2012. Each study area was visited at least once a week to map singing males, pairs and nests. Upon finding a nest, one trail camera (Reconyx PC900 HyperFire Professional High Output Covert; Reconyx, Inc., Holmen, Wisconsin, USA) was installed, pointing directly at the nest entrance at a distance of 1–2 m, depending on topography, and was set up to take 10 images over 10 s per motion detection and one image every 15 min. Trail cameras allowed to survey activity of adults and (old) nestlings, to identify nest predators and to simultaneously monitor many nests, while keeping researcher disturbance at a minimum [78]. Once a week cameras were checked for operational, battery and capacity status until nest success (fledging) or failure (predation, unknown failure) was documented. During the camera checks, nest status was determined (i.e. still active or inactive) and, where applicable, hatchlings were aged with pictures of reference hatchlings from nests where daily visits had been conducted and a reference list given by Wesolowski & Maziarz [74]. A nest was considered successful, when at least one young fledged. In turn, a nest was considered unsuccessful when it was predated (all eggs or hatchlings had disappeared before the expected fledgling date) or when it failed for unknown reasons (eggs still in the nest, but cold to the touch or dead young in the nest without parental activity evident). The exact date of fledging or failure of a nest, and whether it was predated or failed for unknown reasons, was inferred from picture footage. Daily nest survival rates (*dnsr*) and overall nest survival rates were then calculated by taking into account the duration of nest exposure [79], as described in detail under “General statistics” below.

### Evaluated variables in relation to components of reproductive performance

**Fig 1:**
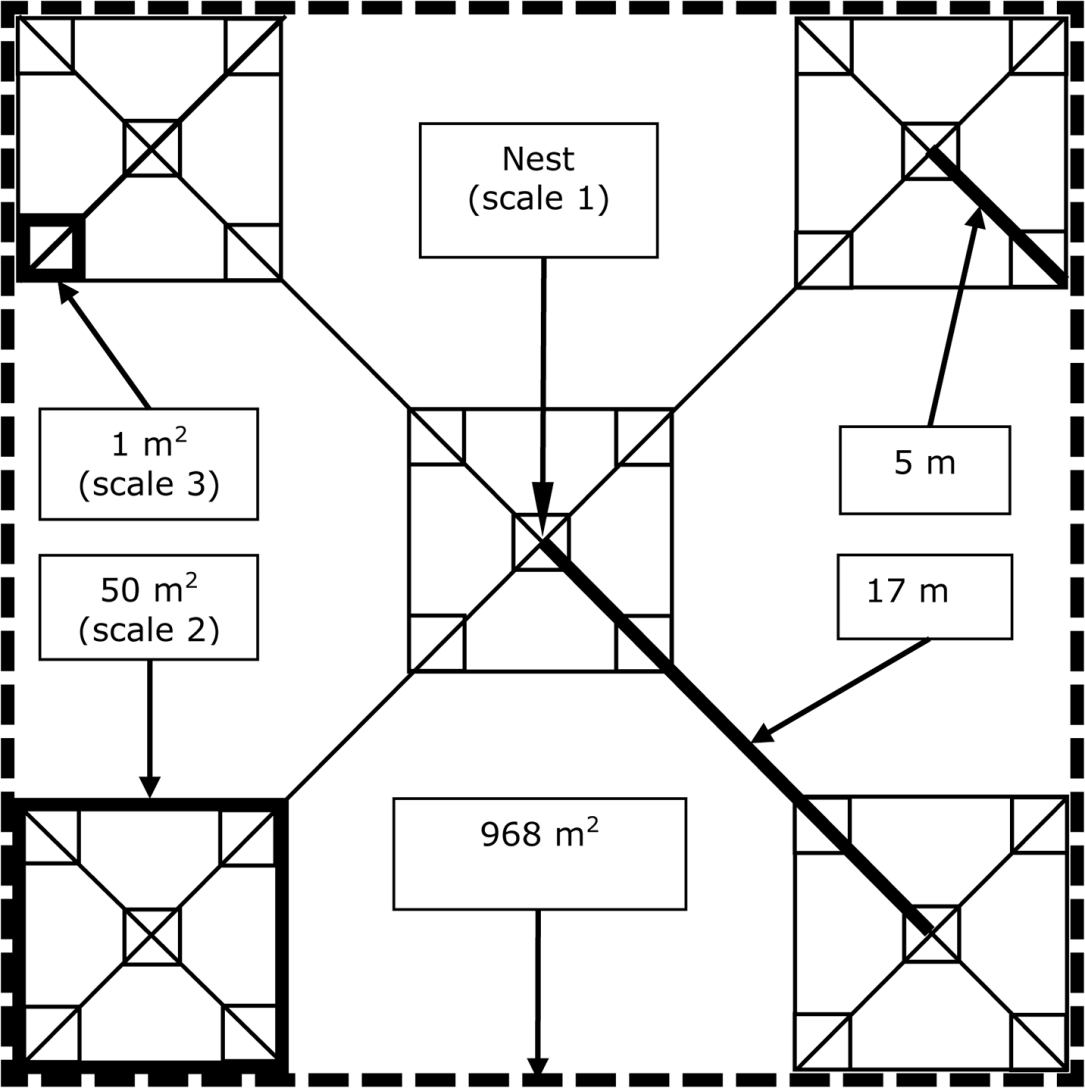
Sampling design with three scales used to map and measure habitat variables and to trap rodents. Scale 1 = nest location (territory center); scale 2 = five 50 m^2^ squares; scale 3 = twenty-five 1 m^2^ squares.

Once a nest had failed or succeeded, variables pertaining to the introduced thematically grouped hypotheses were measured at three sampling scales (Fig 1) within a territory of 968 m^2^, which lies within the reported wood warbler territory size of 500 and 1900 m^2^ [33] Variables evaluated, grouped by hypothesis, were: 1) “start of egg-laying” (timing of breeding hypothesis), 2) “nest age” (temporal exposure hypothesis), 3) “nest concealment index”, “nest location”, “number of bushes”, “number of grass and sedge tussocks”, “vegetation cover” (nest concealment hypothesis), 4) “elevation”, “inclination”, “exposition” (topography hypothesis), 5) “number of trees”, “average tree diameter”, “canopy cover” (tree structure hypothesis), 6) “rodent abundance”, “distance to forest edge”, “distance to trail” (predation and disturbance hypothesis), 7) “distance to closest territory”, “number of territories within 300 m” (density dependence hypothesis), 8) “daily mean temperature”, “total daily rainfall”, “mean temperature”, “mean rainfall”, “rainfall stretch” and “ratio rainfall” (weather hypothesis). Details and descriptions about the variables measured are presented in Table 1.

**Table 1:** Variables used in the modeling of components of reproductive performance in the Swiss wood warbler.

Hypothesis	Variable	Measurement method (description if applicable)	Scale*
Timing of breeding	start of egg-laying	-nest check after 3 days for nests found during building	NA
		-back calculating based on age and nr. of fledglings for nests found after clutch completion assuming one egg laid per day ^#^, 14 days incubation °	
Temporal exposure	nest age	-number of days based on actual or back calculated start of egg-laying	NA
Nest concealment	nest concealment index	-discrete variable from 0 to 5 denoting nest concealment from five viewpoints assessing whether the nest is visible or not from front (entrance hole), back, left, right and top of nest from a distance of 1.5 m	1
		0 = visible from all 5 sides, 5 = not visible from any of the 5 sides	
	nest location	categorical variable with four levels	1
		a) concealed by grass or sedge tussock	
		b) concealed by single plant < 50 cm in height (other than grass/sedge tussock)	
		c) concealed by 2 or more plants < 50 cm in height	
		d) other (including all other concealment possibilities)	
	number of bushes	-number of bushes and young trees > 50 cm in height and < 25.1 cm in stem circumference (territory mean)	2
	number of grass and sedge tussocks	- tussock count (territory mean)	3
	vegetation cover	-vegetation cover in 5%-classes (territory mean)	3
Topography	elevation	-extracted from ecoGIS, http://map.geo.admin.ch/	1
	inclination	-compass inclinometer (territory mean)	2
	exposition	-compass with values from 0 to 360 degrees (territory mean)	2
Tree structure	number of trees	-tree count (territory mean)	2
	tree diameter	-based on circumference at breast height (territory mean)	2
	canopy cover	method described by [80] with adaptions:	2
		-DSLR camera (Nikon D2Xs) with standard lens (18–70 mm f3.5–4.5G ED-IF AF-S DX Zoom Nikkor) and focal length: 35 mm	
		- camera held 1.5 m above ground, pointing vertically up	
		- camera ground plate pointing towards territory center or south east for picture at territory center	
		- Import with raw format in Photoshop CS5	
		- brightness of green and blue colors lowered to minimum and increased to maximum, respectively, to increase contrast	
		-pictures downscaled to 1500 x 1000 pixels and transformed to b/w bitmap	
		- processed by self-written php-script for b/w pixel ratio	
Disturbance	distance to forest edge	- measured in meters in ecoGIS without considering topography	1
	distance to trail	-measured in meters in ecoGIS without considering topography (distance to closest human used trail)	1
Predation risk	rodent abundance	-by live-trapping with longworth traps (Penlon Ltd., Abingdon,+ UK) and “Field Trip Trap Live Catch Trap” (Alana Ecology, Bishops Castle, UK) ^†^	3
		-25 traps per territory for 48 hours with controls every 8 hours	
		-5 traps per 50 m^2^ square (Fig 1) placed without pattern at structures likely used by rodents (e.g. tree, stump, bush, rock, dead wood)	
		-minimum known number alive (mna) used for analysis, due to low recaptures in certain years and/or territories	
Density	distance to	-calculated in meters based on nest coordinates	1
dependence	closest territory		
	number of territories	-number of territories within 300 m of focal territory	1
	within 300 m	-calculated based on nest coordinates	
Weather ^§^ (in relation to *dnsr*)	daily mean temperature	-mean temperature (°C) per day	NA
	total daily rainfall	-mm, between 5:40 a.m. and 5:40 a.m. of the following day	NA
Weather ^§^ (in relation to clutch size and number of fledglings)	mean temperature	-mean temperature (°C) during each of the 3 periods ^±^	NA
	mean rainfall	-mean rainfall during each of the 3 periods ^±^	NA
	rainfall stretch	-longest stretch of days with rainfall during each of the 3 periods ^±^	NA
	ratio rainfall	-ratio of days with rainfall during each of the 3 periods ^±^	NA

* refer to Fig 1 for scale description

^#^ Glutz von Blotzheim et al. 1991

° own data

^†^ see [81] for trap comparison

^§^ all variables derived based on data obtain from SMI-MeteoSwiss stations (Buchs AG, Delemont JU, Glarus GL, Rünenberg BL, Wynau BE, Güttingen TG), which were on average 12.7 km away from study areas

^±^ 1) pre-laying: period of 7 days before egg laying started; 2) pre-incubation: period of 7 days before egg-laying phase, plus the egg-laying phase (clutch size dependent); 3) rearing: period between hatching and fledging.

### Statistical procedures

#### General statistics

We worked with an exposure time method for the analysis of *dnsr* [82]. For nests found before egg-laying, exposure time was the number of days between the actual first-egg date and nest predation or fledging date. For nests found during and after the egg-laying stage, exposure time was the number of days between finding date and fledging or nest predation date. By using trail cameras, the observation interval was always one day and we obtained exact predation and fledging dates. To obtain nest survival rates and standard errors from *dnsr*, we ran a generalized linear mixed-effects model (GLMM) containing the fixed effect “nest age” with values ranging from 1 to 31. 31 days was the average nesting period in our study (5 days egg-laying, 14 days incubation, 13 days rearing). Since incubation typically starts on the same the last egg is laid, we used 31 instead of 32 days for the average nesting period, Using the resulting intercept and estimate from this model, 31 predictor values on the logit scale were calculated through matrix multiplication. The inverse logits of these predictor values resulted in 31 daily nest survival rates. Nest survival rate for the entire nesting period was calculated in two ways: 1) all 31 *dnsr* were multiplied with each other, yielding the primary nest survival rate used in this paper and 2) calculating a mean *dnsr* across the 31 values, then taking this mean *dnsr* to the 31^nd^ power, calculated to compare with Mallord et al. [83]. To obtain survival rates for egg-laying, incubation and rearing stages, respectively, *dnsr* values for each period were multiplied with each other. Standard errors were obtained via calculation of upper and lower confidence intervals in R [84].

#### Model selection

We modeled three components of reproductive performance (clutch size, number of fledglings from successful nests and daily nest survival) using generalized linear mixed effects models (GLMM) in R [84], with the packages lme4 [85], AICcmodavg [86] and arm [87]. For each nest, nest survival was coded and modeled as a binary dependent variable (yes/no) on a day-by-day basis using a logit link function and binomial error structure. Thus, each nest provided multiple data points and we accounted for this dependency with a random effect “nestID”. The dependent variables “clutch size” and “number of fledglings” were modeled assuming a Poisson distribution of errors and a log link function. In all three analyses we included the random effects “study area” and “year” to account for the data dependency arising from using multiple nests per study area and year, respectively. We investigated each thematic hypothesis by building models of all possible combinations with the associated variables (no interactions), including a null model containing only the random effects. As there may be optima for each variable we also included quadratic effects of all variables. Using AICc, Aikake’s Information Criterion [88] corrected for small sample size [89], all models were ranked, taking into account each model’s goodness of fit using its log-likelihood (LL). The model with the lowest AICc was considered the most parsimonious model among all the candidate models examined. A common approach is to also consider models with a ΔAICc < 2 compared to the top ranked model, as they may have equal support to explain variation in the dependent variable [36]. We adopted this approach but added the following two criteria to select models: they had to 1) rank higher than the null model and 2) to have a LL Δ > 1 (compared to models with fewer predictors) or a LL Δ < 1 (compared to models with more predictors) [90]. Through model averaging over all candidate models, we obtained estimates and standard errors from all variables included in models that met the selection criteria described above [91]. All predictor variables with model-averaged estimates larger than their model-averaged SEs were then used in an across-hypothesis analysis (AHA). Here, reproductive performance variables were related to predictor variables pertaining to different hypotheses, using the same GLMM structures as previously outlined. The same model selection approach as described above was applied.

### Ethics statement

All procedures were performed according to the laws of Switzerland and rules of the Swiss Ornithological Institute and approved by the Federal Office for the Environment FOEN (reference # F044-0799) and the Cantonal Office for forest (reference # 410).

## Results

### General findings

Between 2010 and 2012 we found 136 nests of which 57 were classified as predated (42%) and 15 as “unknown failure” (11%). Picture evidence of the predator was available in 84% of the predated nests. Identified nest predators were Eurasian jay (*Garrulus glandarius*, n = 14), stone and pine martens (*Martes foina* and *Martes martes*, n = 13), red fox (*Vulpes vulpes*, n = 11), European badger (*Meles meles*, n = 5), tawny owl (*Strix aluco*, n = 3), domesticated cat (*Felis catus*, n = 1) and Eurasian sparrowhawk (*Accipiter nisus*, n = 1). Across 3 years, nest survival (± SE) over an average nesting period of 31 days was 46.9 ± 0.07% (Table 2) and mean daily nest survival (*dnsr*) 0.976 ± 0.002. Without fox or jay predation (main mammalian and avian nest predators), nest survival over 31 days would have been 57.4 ± 0.1% (mean dnsr: 0.982 ± 0.002) and 55.7 ± 0.08% (mean dnsr: 0.981 ± 0.002), respectively.

**Table 2:** Reproductive performance of Swiss wood warblers in 2010–2012. Shown are means ± SE, with sample sizes in parentheses.

Year	Clutch size	Number of fledglings		Naïve nest success#	Nest survival			
		over successful nests	over all nests		Egg laying	Incubation	Rearing	Whole nesting period*
2010	5.23 ± 0.20 (40)	4.55 ± 0.28 (23)	1.86 ± 0.34 (49)	40.8 (49)	95.5 ± 0.02 (40)	76.9 ± 0.06 (40)	61.5 ± 0.07 (40)	45.2 ± 0.07 (40)
2011	5.55 ± 0.10 (62)	4.71 ± 0.25 (34)	2.46 ± 0.32 (65)	52.3 (65)	99 ± 0.02 (57)	90.6 ± 0.06 (57)	69 ± 0.07 (57)	61.9 ± 0.08 (57)
2012	5.19 ± 0.22 (21)	4.70 ± 0.37 (10)	2.14 ± 0.52 (22)	45.5 (22)	99 ± 0.02 (22)	88.2 ± 0.06 (22)	52.1 ± 0.07 (22)	45.5 ± 0.08 (22)
2010–012	5.38 ± 0.09 (123)	4.66 ± 0.17 (67)	2.19 ± 0.21 (136)	47.1 (136)	96 ± 0.02 (119)	78.5 ± 0.06 (119)	62.2 ± 0.07 (119)	46.9 ± 0.07 (119)

* whole nesting period is based on an average clutch size of 5 eggs, an average of 14 days of incubation and an average of 13 days of rearing.

^#^ Naïve nest success is the proportion of successful nests out of all sampled nests without considering exposure time.

### Daily nest survival rate (*dnsr*)

#### Temporal exposure and timing of breeding

Model selection yielded three models with a ΔAICc < 2 to the top ranked model (ΔAICc of null model = 3.89, Table 3). These models contained the variables “nest age”, “nest age^2^” and “start of egg-laying”. Based on model averaged estimates and SEs (Table 4), only “nest age” (-0.07 ± 0.03) was used for the subsequent AHA.

**Table 3:** Model selection results for the analysis of daily nest survival rate in relation to environmental and social factors.

Hypothesis	Model	K	AICc	ΔAICc	Wt.	LL
Temporal exposure and timing of breeding	nest age	5	312.40	0.00	0.35	-151.17
	nestage^2	6	313.28	0.88	0.22	-150.60
	nest age + start of egg-laying	6	314.34	1.94	0.13	-151.13
	…					
	null model	4	315.22	3.89	0.05	-154.13
Nest concealment	nr of tussocks	5	312.75	0.00	0.17	-151.35
	nr of tussocks + concealment index	6	313.54	0.79	0.12	-150.73
	nr of tussocks + nr of bushes	6	313.6	0.81	0.12	-150.74
	nr of tussocks^2	6	313.65	0.91	0.11	-150.79
	nr of tussocks^2 + nr of bushes	7	314.02	1.27	0.09	-149.96
	nr of tussocks^2 + concealment index	7	314.7	1.91	0.1	-150.28
	nr of tussocks + nr of bushes + concealment index	7	314.7	1.93	0.07	-150.29
	…					
	null model	4	316.29	3.55	0.03	-154.13
Weather	null	4	316.29	0.00	0.30	-154.13
	rainfall	5	316.35	0.06	0.29	-153.15
	temperature + rainfall	6	318.03	1.74	0.1	-152.98
	temperature	5	318.18	1.88	0.12	-154.06
Topography	null	4	316.29	0.00	0.13	-154.13
	exposition^2	6	316.54	0.24	0.12	-152.23
	exposition	5	316.89	0.59	0.10	-153.42
	exposition^2 + inclination	7	317.30	1.01	0.08	-151.60
	inclination	5	317.78	1.49	0.06	-153.87
	inclination^2 + exposition^2	8	317.80	1.51	0.06	-150.84
	elevation	5	318.18	1.89	0.05	-154.06
	expsistion^2 + elevation	7	318.29	2.00	0.05	-152.10
Tree structure	null	4	316.29	0.00	0.18	-154.13
	nr of trees	5	317.22	0.92	0.12	-153.58
	average tree diameter	5	317.93	1.64	0.08	-153.94
	canopy cover^2	6	317.98	1.69	0.08	-152.95
	canopy cover	5	318.13	1.84	0.07	-154.04
Predation risk	null	4	316.29	0.00	0.66	-154.13
and	nr of rodents	5	318.29	1.99	0.24	-154.12
Disturbance	null	4	316.29	0.00	0.34	-154.13
	distance to forest edge	5	317.55	1.26	0.18	-153.75
	distance to closest path	5	317.78	1.49	0.16	-153.87
Density dependence	null	4	316.3	0	0.3	-154.13
	nr of territories within 300m	5	317.62	1.33	0.17	-153.79
	nr of territories within 300m^2	6	317.98	1.69	0.14	-152.96
	distance to closest territory	5	318.14	1.84	0.13	-154.04
Across- hypothesis- analysis	nr of tussocks + nest age	6	307.95	0.00	0.54	-147.94
	nr of tussocks + nest age + concealment index	7	309.27	1.33	0.28	-147.59
	…					
	null model	4	316.29	8.35	0.01	-154.13

Models are separated into thematically grouped hypotheses, including an across-hypothesis-analysis at the end. Models with a ΔAICc < 2 to the highest-ranked model as well as null models are presented. K = number of parameters in the model (only fixed effects are shown in the table). Wt. = Akaike’s weight; LL = Log likelihood of a model. “…” refers to additional models examined, but not listed in detail to avoid overlong table, as they were little informative. N = 115.

**Table 4:** Model-averaged estimates and standard errors (SE) based on all models per hypothesis group.

Hypothesis	Variable	Model averaged estimate	Model averaged SE
Temporal exposure	nest age	-0.07	0.03
	nest age (linear term)	0.03	0.10
	nest age (quadratic term)	0.00	0.00
timing of breeding	start of egg-laying	0.01	0.02
Concealment	nr tussocks	0.62	0.34
	concealment index	0.17	0.16
	nr bushes	0.22	0.23
	nr tussocks (linear term)	0.27	0.48
	nr tussocks (quadratic term)	0.43	0.49
Weather	mean daily temperature	0.08	0.17
	total daily rainfall	0.35	0.28
Across hypotheses	nr of tussocks	0.76	0.41
	nest age	-0.07	0.03
	concealment index	0.18	0.16

Only estimates and SE for variables in the top-ranked model and in models with ΔAICc < 2 to this one are shown. For the hypothesis groups topography, tree structure, predation risk, disturbance and density dependence, estimates and standard errors were not averaged as there were no models ranked better than the null model (see methods for variable exclusion criteria). Estimates (and SE) for the variables of these hypothesis groups are therefore not shown.

#### Nest concealment

Model selection yielded six models with a ΔAICc < 2 to the top ranked model (ΔAICc of null model = 3.94). These models contained the variables “number of grass and sedge tussocks”, “number of grass and sedge tussocks^2^”, “number of bushes” and “concealment index”. Based on model averaged estimates and SEs, “number of grass and sedge tussocks” (0.62 ± 0.34) and “nest concealment index” (0.17 ± 0.16) were used for subsequent AHA (Tables 3 and 4).

#### Weather

While model selection classified the null model as the highest ranked model, it is worth noting that one model had a ΔAICc very close to the null model (ΔAICc = 0.06) suggesting that total daily rainfall may well have an influence on *dnsr*. The model averaged estimate and SE for total daily rainfall was 0.35 ± 0.28. However, abiding by our selection criteria, we did not analyze total daily rainfall together with other variables in the AHA.

#### Topography, tree structure, predation risk, disturbance, density dependence

In all five thematic hypothesis groups, the respective null models were always ranked highest (Table 3) and therefore none of the variables were considered in the AHA.

#### Across hypotheses analysis

**Fig 2:**
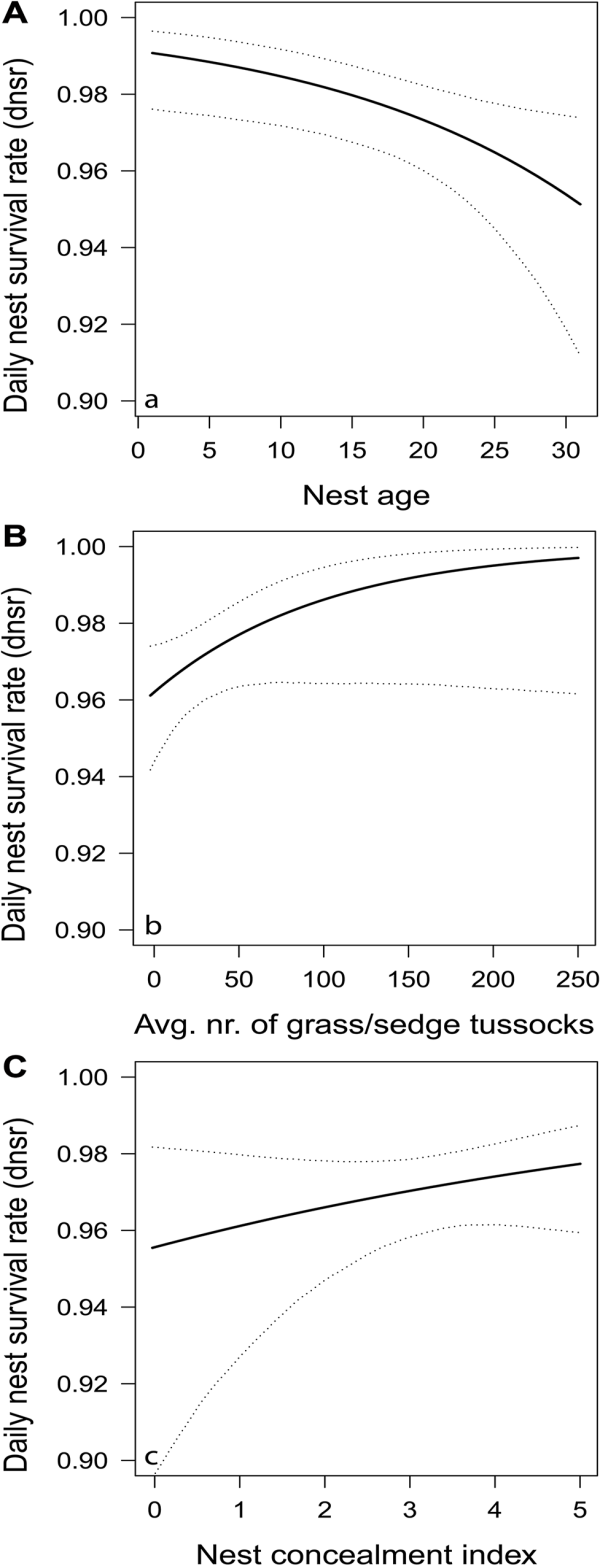
Daily nest survival rate in relation to a) nest age (in days), b) number of grass and sedge tussocks in a given territory, and c) nest concealment. Plots show fitted values (solid lines) and 95% confidence intervals (dashed lines) based on a model that includes nest age, number of grass and sedge tussocks and nest concealment (Table 3, AHA results, n = 115).

Model selection for the AHA yielded one model with a ΔAICc < 2 to the top ranked model (ΔAICc of null model = 8.35; Table 3). The top ranked model contained the variables “number of grass and sedge tussocks” and “nest age”, the second-best model “number of grass and sedge tussocks”, “nest age” and “nest concealment”. Based on model averaged estimates and SEs, *dnsr* was negatively related to “nest age” (-0.07 ± 0.03, Fig 2A, Table 4) and positively related to “number of grass and sedge tussocks” (0.76 ± 0.41, Fig 2B, Table 4) as well as to “nest concealment” (0.18 ± 0.16, Fig 2C, Table 4).

### Clutch size and number of fledglings

**Fig 3:**
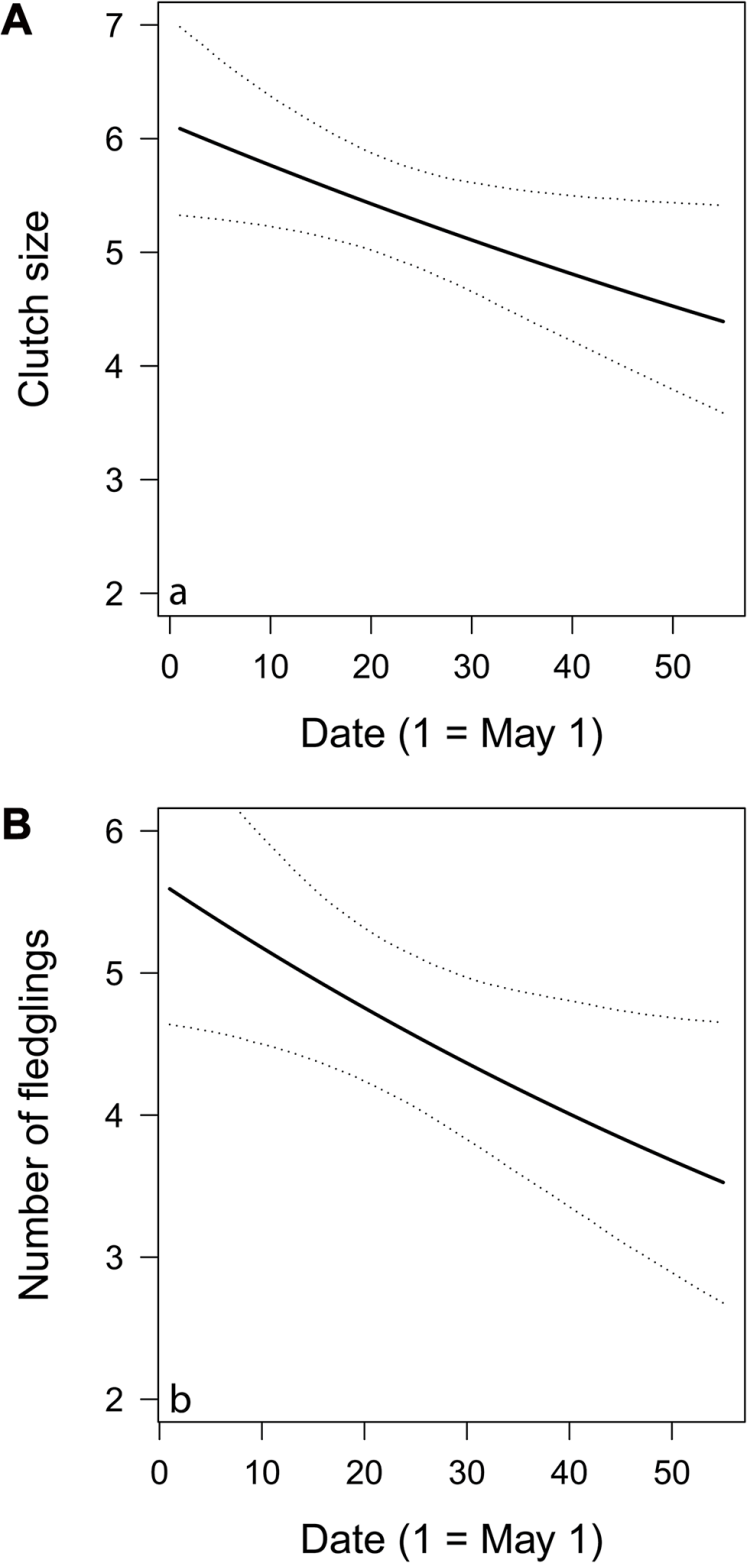
a) Clutch size (n = 115 nests) and b) number of fledglings (n = 64 nests) plotted against start of egg laying. Data were pooled over the three years of study. Plots show fitted values (solid lines) and 95% confidence intervals (dashed lines) based on the highest ranked model including only start of egg-laying in both panels.

Model selection for both clutch size and number of fledglings resulted in one top-model containing only “Start of egg-laying” (ΔAICc to null models: 2.48 for clutch size, 2.82 for number of fledglings). Estimates (and SE) for “Start of egg-laying” were -0.01 ± 0.00 in both analyses of clutch size (Fig 3A) and number of fledglings (Fig 3B). In the analyses of all other thematic hypotheses groups, the null models were always ranked highest and therefore no other variables were considered nor was an AHA necessary.

## Discussion

Reproductive performance of the wood warbler in our study area was associated to temporal exposure, timing of breeding and concealment of nests. Daily nest survival was positively related to the number of grass and sedge tussocks present in a given territory, nest concealment and nest age. Moreover, both clutch size and number of fledglings decreased, the later in the season egg-laying started.

### Nest predation

Clutch sizes, nest survival, nest success and number of fledglings of Swiss wood warblers seem to be lower than in most other studies [64,74,83,92,93]. Nest success and nest survival of wood warblers in Switzerland were furthermore lower than in other ground-nesting forest passerines of Europe [94,95,96] and North America [97] (Table 5). In our study, the primary cause of nest failure was nest predation, amounting to 79% of all failures (n = 72). Increased predation pressure by foxes due to increasing fox populations has been discussed to contribute to the decline of wood warblers, but our data does not lend support to this suspicion. Omitting nests predated by foxes from our statistical calculations does result in higher nest survival over the three study years, but nest loss rate was still around 43%. Also, nest survival was higher in 2010 (year with most fox predation, n = 9 nests) than in 2012 (year with no fox predation). Indeed, the main predator of wood warbler nests in Switzerland was the Eurasian jay, similar to observations from Welsh oakwoods [83], where jays and birds of prey were responsible for 93% of all predation events, with only two cases of predation by mammals. Mammals in Switzerland caused as much as 52.6% of all predatory events, suggesting that predator guilds differed between the two regions.

**Table 5:** Comparison of reproductive performance parameters of wood warblers across Europe and of other ground nesting forest birds of Europe and North America.

Study	Number of study years; country	Mean clutch size (sample size)	Mean number of fledglings per successful nest	Mean number of fledglings per nest	Naïve nest success in % (sample size)	Nest survival using exposure time methods in % (sample size)
**Wood Warbler**						
This study	3; Switzerland	5.38 ± 0.09 (123)	4.66 ± 0.17 (64)	2.19 ± 0.21 (136)	47.1 (136)	46.9* ± 0.07 (119)
						47.1° ± 0.02 (119)
Herremans 1993	5; Belgium	-	5.64	-	-	-
Hillig 2009	1; Germany	5.77	4.77	2.63	57.35 (68)	-
Hölzinger 1999	-; Germany	5.84 (583)	-	3.3	-	-
Lippek 2009	5; Germany	5.36 ± 0.09	-	-	-	-
Mallord et al. 2012	3; Wales UK	-	-	-	-	50.7 ^†^ ^#^ (167)
Moreau 2001	5; France	5.6	5.36	3.16	59 (122)	-
Reinhardt 2003	1; Germany	6.11 ± 0.09	-	-	50 (5)	-
Wesolowski & Maziarz 2009	8, Poland	6.32	-	-	34.6 ^#^	-
**other species**						
Wesolowski & Tomialojc 2005	Poland					
- *Phylloscopus collybita*	5	-	-	-	44 (169)	-
- *Troglodytes troglodytes*	4	-	-	-	40 (101)	-
Hölzinger 1999	Germany					
- *Phylloscopus bonelli*		4.9 (103)	-	2.86	-	-
- *Phylloscopus collybita*		5.24 (617)	-	2.48	-	-
- *Phylloscopus trochilus*		5.65 (77)	-	3.64	-	-
- *Erithacus rubecula*		5.14 (715)	-	3.64	-	-
Yanes & Suarez 1995	Iberian peninsula					
- *Erithacus rubecula*	12	-	-	-	-	61.2 (47) ^#^
- *Luscinia megarhynchos*	13	-	-	-	-	74.8 (32) ^#^
- *Phylloscopus collybita*	10	-	-	-	-	75.9 (88) ^#^
Martin 1993						
*Oreothlypis celata*	-	-	-	-	66.7 (90)	50.1 ^#^
*Oreothlypis virginiae*	-	-	-	-	69.2(26)	58 ^#^
*Cardellina rubrifrons*	-	-	-	-	60 (30)	51.8 ^#^
*Junco hyemalis*	-	-	-	-	69.1 (55)	47 ^#^
*Mniotilta varia*	-	-	-	-	73.7 (19)	49.9 ^#^
*Helmitheros vermivorum*	-	-	-	-	78.6 (14)	71 ^#^
*Seiurus aurocapilla*	-	-	-	-	71.4 (14)	48.9 ^#^

Standard errors and sample sizes are provided, when extraction from the original publications was possible.

* multiplying all 31 *dnsr* values with each other (31 was the mean duration of the nesting period of wood warblers in Switzerland in this study).

° using a daily nest survival rate of 0.976 ± 0.002 and an exponent of 31 (this study).

^†^ using a daily nest survival rate of 0.979 ± 0.003 [83] and an exponent of 31 (this study).

^#^ values from data sets with nest losses only due to predation.

Rodents are known to be important in territory selection of wood warbler [49,50]. Furthermore, rodents have been documented to predate nests of several bird species [98,99] and have been suspected to predate wood warbler nests as well [74,100,101]. Apart from one instance where a red squirrel (*Sciurus vulgaris*) killed but did not eat all nestlings of a nest, there is no picture evidence of any single event of direct predation by smaller rodents such as *Apodemus* or *Myodes* species in our study or elsewhere [83]. This is not due to a problem of detection as the photo footage revealed several occasions of rodents inspecting nests.

### Temporal exposure

Higher nest survival during incubation than during rearing is documented in several studies [16,20,36,102]. The negative relationship observed between *dnsr* and nest age most likely reflects a change in parental activity at the nest. The number of visits to the nest by parents dramatically increases from incubation to chick rearing due to the intense food provisioning [33], which will in turn augment nest predation risk [29]. In wood warblers, chick provisioning peaks on approximately day 9, with up to 650 adult visits in 15 h (roughly 43 times per h), compared to the incubation period when foraging trips by the female amount to once or twice per hour [33]. Given the huge energy and protein demand of rapidly growing nestlings, parents cannot limit the cues inadvertently given to predators [29], thus essentially relying on nest concealment to overcome nest predation. Moreover, with advancing age, begging nestlings may further augment the risk of a nest being visited by predators [103]. Finally, nest predation rates may also increase simply due to a temporal synchronization of the reproductive cycles of both predators and wood warblers: adult predators seek food to rear their own demanding young while early dispersing juvenile predators suddenly increase the number of predators present in wood warblers’ environment [36].

### Timing of breeding

We found a negative relationship between both clutch size and number of fledglings and “start of egg-laying”. Decreasing reproductive performance as the season progresses is a pattern found in many bird species [17,18,19,21]. As we never recorded partial predation, seasonal declines of clutch size and number of fledglings were mediated through factors pertaining to either the date or the quality hypotheses. According to the quality hypothesis, seasonal declines in reproductive performance occur due to late breeding individuals being of lower quality due to inexperience (e.g. first year breeders), worse physiological condition (senescent or injured individuals), or generally low foraging, predation avoidance or nest building skill compared to early breeding individuals. According to the date hypothesis, in contrast, a seasonal decline of reproductive performance may occur due to deteriorating environmental conditions for breeding as the season advances, such as worsening weather conditions, decreasing food availability and/or accessibility, or increasing predation pressure [104,105]. Disentangling the two hypotheses is not possible with our data, but would require experiments, as conducted for example by Verhulst et al. [105] on great tits (*Parus major*) and Christians et al. [104] on European starlings (*Sturnus vulgaris*).

Food availability is not only intricately embedded in the two above hypotheses but also drives habitat selection of birds in general. In the wood warbler, the importance of food availability remains unclear, however. While Wesolowski and Maziarz [74] found a positive correlation between abundance of wood warblers and caterpillars (main food source) [33], Herremans [64] did not. The link between caterpillar abundance and reproductive success, while unclear for the wood warbler, has been shown for other bird species, such as great tits (*Parus major*) [18]. However, caterpillars are not the sole food source for birds such as wood warblers or great tits. Great tits select spiders early in the breeding season, which may breed more than a month earlier than the wood warbler [106], and exhibit prey switching once caterpillars reach body masses around 10–12 mg [107]. Wood warblers may exhibit similar behavior where early breeding individuals attempt to match hatching of young with caterpillar peak abundance and late breeding individuals switch to spiders and/or other prey items as caterpillar abundance declines [108]. Hence, wood warblers appear to exhibit very flexible foraging behavior and are unlikely to track only few specific food sources.

### Nest concealment

Nest site selection and nest concealment influence nest survival in many bird species [21,29,35]. In the wood warbler, nest survival increased with nest concealment and availability of grass and sedge tussocks. The availability of grass and/or sedge tussocks alone is however not sufficient as they also have to be accessible for wood warbler, which is not the case if a shrub layer is present. Not only direct nest concealment seems to be important, but also the dominant ground vegetation surrounding the nest, with which successful nests appear to blend in. Thus, the existence of sufficient patches of grass and sedge appears to be an essential habitat requirement of the wood warbler. The positive correlation between *dnsr* and nest concealment may explain why only 31% of nest predation occurred during egg-laying and incubation, when parental activity around the nest is very limited and a concealment effect may fully unfold, compared to the rearing stage, when parental activity peaks and the majority (69%) of predation events took place (z = 2.5, p-value = 0.01). Martin et al. [29] have shown that increasing parental activity at the nest can act antagonistically to nest concealment. Nests in the incubation stage might hence benefit most from nest concealment when the limited activity of the brooding females does not attract nest predators. Circumstantial evidence from our nest cameras for the incubation period suggests that mammalian predators mostly became aware of nest presence after inadvertently flushing the incubating female. We also assume that visually hunting predators like jays became aware of nests by observing feeding parents and not through detecting the nest itself.

### Weather

The lack of evidence for a link between our reproductive performance variables and weather circumstances may have resulted from rather homogenous weather conditions throughout the breeding season without severe weather events such as prolonged cold, heat, rain or drought spells (Sergio 2003). Alternatively, predation may have been so strong that it masked weather effects.

### Topography, tree structure, predation risk, disturbance, density dependence

Variables pertaining to topography, tree structure, predation risk and disturbance received no or only marginal support for an effect on reproductive performance. While several models ranked within a ΔAICc < 2 to the top ranked model, the null model was always ranked highest in all five groups. In the wood warbler, variables related to topography, tree structure and predation risk have been shown to strongly affect territory settlement within forest stands [49,50,109,110]. Differences pertaining to aforementioned variables between successful and unsuccessful territories may thus become negligible in later phases, such as reproduction. As nest abandonment seemed to have played a marginal role in our study, it is questionable whether disturbance, notably of anthropogenic origin, impacts reproductive success at all. Nests built close to hiking trails were not less successful than nests away from hiking trails (x^2^ = 0.203, p-value = 0.65): nests built at > 11 m (n = 115 of 136 nests) and 1–10 m (n = 21 of 136 nests) from trails had a survival rate of 54.6% and 42.9%, respectively. Only one nest closer than 1 m from a trail was classified as “unknown failure”, i.e. might be attributed to direct human disturbance.

There was no evidence for an effect of density on reproductive performance: models with the two density indices were far worse supported than the null model. We had expected that at least one of the components of breeding output would be linked to density. Further studies are needed to understand the reasons of territory clustering reported in wood warbler populations elsewhere [64].

### Conclusions and consequences for species conservation

Using trail cameras, we were able to identify nest predators in 84% of all predation events, to accurately document the dates of nest predation or fledging and to estimate daily nest survival rates. By visiting our cameras only two or three times during the entire nesting period, we decreased the risk that the observer interferes with breeding, hence minimizing any observer bias in our data.

Although predation is the principal cause of nest failure in Swiss wood warblers, it is not yet possible to draw any conclusions about the potential impact of nest predation on the species’ demographic trends. An appropriate assessment of whether mortality via nest predation is (fully or partially) additive or compensatory would necessitate predator exclosure experiments. Therefore, it would be premature to claim that protecting nests against predators might be an option to improve the demographic status of the wood warbler in Switzerland, especially given the nomadic nature of the species [49].

That reproductive performance of Swiss wood warblers is strongly related to the availability and especially accessibility of grass and sedge tussocks is a major finding of the present study and, combined with results on territory selection [50,111], provides essential evidence-based guidance for planning forestry interventions that can benefit the species. Nevertheless, the implementation of our finding is not as straight forward as simple single tree removal to promote growth of the field layer. Depending on topographical factors (e.g. slope inclination or site elevation) and factors such as soil composition, grasses and sedges would grow, but so would other field layer vegetation (e.g. *Rubus* species or various shrubs), hence potentially decreasing habitat suitability for the wood warbler [50,111]. As there is evidence that wood warblers require large areas to allow for clustered settling of multiple individuals [64], conservation measures should aim at increasing the area of relatively homogenous forest stands featuring suitable habitats characterized by abundant grass and sedge tussocks (this study), high tree numbers, few bushes and an intermediate ground vegetation cover [50] as well as a relatively closed canopy [112]. Such conditions are typically found in forest stands of middle age (i.e. pole wood) in managed forests.
